# Construction of a Conditionally Asporogenous *Bacillus thuringiensis* Recombinant Strain Overproducing Cry Protein by Deletion of the *leuB* Gene

**DOI:** 10.3389/fmicb.2020.01769

**Published:** 2020-07-24

**Authors:** Meifang Quan, Jinli Peng, Zirong Zhu, Pengji Zhou, Sisi Luo, Junyan Xie, Liqiu Xia, Yunjun Sun, Xuezhi Ding

**Affiliations:** ^1^Hunan Provincial Key Laboratory of Microbial Molecular Biology, State Key Laboratory of Developmental Biology of Freshwater Fish, College of Life Science, Hunan Normal University, Changsha, China; ^2^Key Laboratory of Molecular Epidemiology of Hunan Province, School of Medicine, Hunan Normal University, Changsha, China

**Keywords:** LeuB, *Bacillus thuringiensis*, sporulation, quantitative proteomics, metabolic regulation

## Abstract

One of the common shortcomings with *Bacillus thuringiensis* (Bt) biopesticides in field application is their instability under UV irradiation. In Bt, the *leuB* gene encodes the 3-isopropylmalate dehydrogenase. In addition to its role in leucine biosynthesis, LeuB would be likely recruited to catalyze the dehydrogenation of malate in the final step of tricarboxylic acid cycle during sporulation. In this study, we constructed a Bt recombinant strain in which the gene *leuB* was deleted by using the markerless gene deletion system. The Δ*leuB* mutant strain showed a conditionally asporogenous phenotype while overproducing insecticidal crystal proteins and retaining its insecticidal activity well in both fermentation and LB media. Furthermore, the metabolic regulation mechanisms of LeuB was elucidated by iTRAQ-based quantitative proteomics approach. Evidences from proteomics data suggested that the inhibited supply of pyruvate (carbon source) was an important factor related to the conditionally asporogenous feature of the mutant. Consistently, the mutant regained its ability to sporulate in LB medium by adding 1% glucose or 1% sodium pyruvate. Taken together, our study demonstrated that deletion of the *leuB* gene resulted in delayed or completely blocked mother cell lysis, allowing the crystals encapsulated within cells, which makes this recombinant strain a good candidate for developing Bt preparations with better UV-stability.

## Introduction

*Bacillus thuringiensis* (Bt) is an entomopathogenic bacterium that produces large amounts of insecticidal crystal proteins (ICPs, including Cry, and Cyt protoxins) during sporulation (Melo et al., 2016). It forms dormant endospores in response to nutrient deprivation, and spores can further re-enter the vegetative cell life cycle when encounter conditions favorable for germination (Paredes-Sabja et al., 2011; Setlow et al., 2017). The environmental stability and resistance properties of Bt spores are attributed to their special structure and chemical components. It has been reported that the protective small polar molecule dipicolinic acid (DPA) is a major component in spores of *Bacillus* and *Clostridium* species, comprising 5–15% of the spore dry weight. DPA is mostly responsible for dehydration of the spore, which is associated with resistance to heat or desiccation (Magge et al., 2008; Orsburn et al., 2010).

On the other hand, the expression of Cry proteins in the mother cell of Bt is intimately coupled to sporulation. That is because in addition to being involved in spore development, the sporulation-specific sigma factors σ^E^ and σ^K^ are also essential for transcription of most *cry* genes (Bravo et al., 1996; Schnepf et al., 1998; Yang et al., 2012). *cry1* and *cry2* are the typical sporulation-dependent genes that require the binding of σ^E^ and (or) σ^K^ to the specific sequence of their promoter regions to start the transcription (Schnepf et al., 1998). Therefore, overexpression of sporulation-dependent *cry* genes begins in stage III of sporulation and continues through stage VII, accumulating as crystalline inclusions within the mother cell. The Cry toxins are toxic against many kinds of pest larvae, which makes Bt the most successful microbial pesticide worldwide (Roh et al., 2007; Jouzani et al., 2017).

The commercial Bt biopesticides are mixtures containing spores and insecticidal crystals. Although Bt is closely related to its more dangerous relatives such as *Bacillus anthracis* and *Bacillus cereus sensu stricto*, and available data have demonstrated the long-term persistence of Bt spores in soil, there is no direct evidence to suggest that Bt can cause disease in humans, and the safety of Bt pesticides is supported by decades of safe use history (Van Cuyk et al., 2011; Hendriksen and Carstensen, 2013; Biological Hazards Panel of EFSA, 2018). However, despite their advantages in many aspects such as high specificity and low ecotoxicity, the Bt products still need some improvement, especially in terms of potency. Because the active ingredient ICPs have poor environmental stability and could degrade quickly under UV radiation, the duration of pest control for Bt products is relatively shorter comparing with the traditional synthetic insecticides (Myasnik et al., 2001; Yang et al., 2018). Therefore, how to protect the crystal proteins from UV deactivation is becoming a concern for scientists. In previous studies, it was shown that the sporulation-deficient *spo0A* or σ^k^ deletion Bt mutant strains produced normal insecticidal crystals encapsulated in the mother cell protected from UV degradation (Lereclus et al., 1995; Sanchis et al., 1999). However, a drawback of the *spo0A* deletion recombinant Bt strains is that the expression of sporulation-dependent *cry* genes was seriously inhibited and must be located under the control of the sporulation-independent *cry3A* gene promoter. Recently, another study showed that deletion of the cell wall hydrolase gene *cwlC* in Bt strain completely blocked the mother cell lysis without impacting sporulation or ICPs production (Chen et al., 2018). These aforementioned studies indicate that the genetic engineering approach provides a promising strategy for developing new recombinant Bt insecticides with better environmental stability.

The *leuB* gene encodes the 3-isopropylmalate dehydrogenase, an enzyme in the leucine biosynthesis pathway (Graczer et al., 2013). LeuB has been confirmed to have a very broad substrate specificity and can catalyze the dehydrogenation of malate (Kawaguchi et al., 1993; Drevland et al., 2007). A previous study reported that the expression of malate dehydrogenase and its two isoenzymes were severely inhibited, whereas the expression of LeuB was significantly up-regulated during sporulation in Bt. In addition to its role in branched chain amino acid (BCAA) biosynthesis, LeuB is probably used for malate dehydrogenation in the final step of tricarboxylic acid (TCA) cycle during sporulation (Wang et al., 2013). Therefore, it is speculated that LeuB is intimately involved in the metabolic regulation related to sporulation.

We report herein the construction and phenotype characterization of a *leuB* gene knockout strain that shows a conditionally sporulation-deficient phenotype with delayed or completely blocked mother cell lysis, while over-producing large amounts of ICPs. By using iTRAQ-based quantitative proteomic approaches, we further presented evidences revealing the important role of LeuB in the metabolic regulation network related to sporulation and ICPs accumulation.

## Materials and Methods

### Bacterial Strains and Culture Conditions

The strains used or constructed are listed in Table 1. *Escherichia coli* DH5α was used for routine cloning and plasmid construction. *E. coli* cells were cultured at 37°C in lysogeny broth (LB) medium (tryptone 10 g/l, yeast extract 5 g/l, NaCl 10 g/l) supplemented with appropriate antibiotics as needed at a final concentration of 100 μg/ml for ampicillin, 100 μg/ml for spectinomycin and 50 μg/ml for kanamycin. For Bt strains, single colonies on LB agar plate were firstly inoculated into LB medium for overnight culture with shaking at 30°C and then subsequently subcultured 1:100 to the fermentation medium (glucose 18 g/l, tryptone 14.5 g/l, K_2_HPO_4_ 2.5 g/l, FeSO_4_⋅7H_2_O 0.02 g/l, MnSO_4_⋅ H_2_O 0.02 g/l, MgSO_4_⋅7H_2_O 0.25 g/l), nutrient sporulation medium (Malvar et al., 1994) (NSM; nutrient broth 8 g/l, MnCl_2_ 0.05 mM, CaCl_2_ 0.7 mM, MgCl_2_ 1.0 mM) or 1/2 LB medium (tryptone 5 g/l, yeast extract 2.5 g/l, NaCl 5 g/l).

**TABLE 1:** Bacterial strains and plasmids.

Strains/plasmids	Characteristics	Source or References
**Strains**		
DH5α	*E. coli*, host for routine cloning	Lab store
Bt 4.0718	Bt wild type strain(CCTCC No.M200016)	Lab store
Bt Δ*leuB*	Markerless *leuB* gene deletion mutant of Bt 4.0718	This work
Bt Δ*leuB*:*leuB*	Genetically complementary strain of Bt Δ*leuB* with pRPleuB plasmid	This work
**Plasmids**		
pRP1827	Helper plasmid for conjugative transfer; Amp^R^	Janes and Stibitz, 2006
pRP4332	Bt-*E. coli* shuttle plasmid; Km^R^; containing I-SceI restriction enzyme encoding gene	Janes and Stibitz, 2006
pRP1028	Bt-*E. coli* shuttle plasmid; Spc^R^; containing temperature-sensitive suicide *Bt* replicon and an I-SceI recognition site, etc.	Janes and Stibitz, 2006
pRP1028-leuB	pRP1028 carrying upstream homologous arm of *leuB* (*UleuB*) and downstream homologous arm of *leuB* (*DleuB*)	This work
pRPleuB	pRP1028 carrying *Perm*-*leuB* fragment	This work

### Construction of Mutant Strains

All plasmids used are shown in Table 1. All primers used are presented in Supplementary Table S1. pRP1028 was an *E. coli*-Bt shuttle plasmid with a temperature-sensitive Bt replicon and I-SceI recognition site. To construct the plasmids for *leuB* deletion, a 682-bp upstream region and a 655-bp downstream region flanking *leuB* were amplified from the Bt 4.0718 genomic with the UleuB-F/UleuB-R and DleuB-F/DleuB-R primer pairs, respectively. The generated pair of upstream and downstream homologous arms flanking *leuB* were further inserted into plasmid pRP1028 through the *Bam*HI/*Sal*I and *Sal*I/*Kpn*I sites, respectively. The final recombination plasmid was named as pRP1028-leuB.

The *leuB* deletion strain was developed through a homing endonuclease I-SceI mediated markerless gene replacement system (Janes and Stibitz, 2006). The detailed procedures were performed strictly according to the method described previously (Janes and Stibitz, 2006; Wang et al., 2016). Briefly, the gene deletion plasmid pRP1028-leuB was introduced into the recipient strain Bt 4.0718 through a triparental mating conjugative transfer procedure using *E. coli* DH5α (containing pRP1028-leuB) as the donor strain and *E. coli* DH5α (containing the helper plasmid pSS1827) as the helper strain. Then the whole pRP1028-leuB plasmid was integrated into the Bt 4.0718 chromosome by the first homologous recombination. The correct recombinant strain integrated with plasmid was selected and further transformed with the I-SceI expression plasmid pRP4332 through the second conjugative transfer experiment. The homing endonucleases I-SceI recognized the 18 bp unique site within the integrated plasmid and resulted in chromosomal double-stranded breaks, which stimulated the second homologous recombination. Consequently, approximately 50% would have undergone the desired gene deletion. The correct *leuB* deletion strains were verified by PCR and DNA sequencing with primer pairs UleuB-F/DleuB-R and leuB-F/leuB-R (amplifying the *leuB* ORF).

For complementation of Bt Δ*leuB*, a 1,464-bp fragment comprising the promoter of erythromycin resistance gene (*Perm*) and the *leuB* ORF was amplified by SOE PCR using primers listed in Supplementary Table S1. Briefly, the *Perm* was amplified with primer pairs P-F/P-R, and the *leuB* ORF was amplified with primers E-F/E-R. These two PCR fragments were mixed and annealed, and were used as template for the amplification of the fusion fragment *Perm-leuB* using primer pairs P-F/E-R. Finally, the *Perm-leuB* fragment was cloned into pRP1028 through the *Bam*HI/*Sal*I site, and the resulting construct pRPleuB was conjugated into Bt Δ*leuB*. The correct complemented strain Bt Δ*leuB*:*leuB* was verified by PCR and sequencing.

### Sporulation and Heat-Resistance Assays

Strains were cultured in 30 ml of LB or fermentation liquid medium at 30°C with shaking at 200 rpm. One milliliter samples were collected at T_0_ (the end of the exponential phase) and T_34_ (spore-release period), respectively. The number of vegetative cells was estimated by counting the colony-forming units (CFUs) on the LB or fermentation medium plates. The number of spores was determined by counting the heat-resistant (65°C for 40 min) CFU on plates (the unlysed cells of Bt Δ*leuB* group needed sonication disruption to release the spores prior to heat treatment). Sporulation efficiency was calculated as described previously (Chen et al., 2018). To perform the relative heat-resistance assay quantitatively, 30 μl samples of each group were heated at 90°C for 15 min, and then serially diluted for CFU determination.

### RNA Isolation and qRT-PCR Analysis

For qRT-PCR analysis of gene expression, total RNA was prepared from the bacterial cells harvested at indicated time point using TRIzol Reagent (Invitrogen Biotechnology Co., Ltd., Shanghai, China). The cDNA synthesis was carried out using a RevertAid^TM^ First Strand cDNA Synthesis Kit according to the manufacturer’s procedure. The qRT-PCR analysis was performed with Power SYBR^®^ Green PCR Master Mix in an Applied Biosystems 7500 real-time PCR system (Applied Biosystems, United States). The specific primers used for qRT-PCR were listed in Supplementary Table S1. All genes were normalized to the 16S rRNA gene. Fold changes for target genes from Bt Δ*leuB* relative to that from Bt 4.0718 was determined using the threshold cycle (2^–ΔΔ*CT*^) method.

### Transmission Electron Microscopy

The Bt 4.0718 and Bt Δ*leuB* strains were collected at 24 and 48 h, respectively. After centrifugation (12,000 rpm, 4°C, 2 min), the cell pellets were fixed with 2.5% glutaraldehyde in 0.2 M phosphate buffer (pH7.4) at 4°C for overnight. After washing three times with 0.1 M PBS buffer, cells were firstly fixed with 1% tannin solution at 4°C for 2–3 h, washed with PBS buffer for another three times, and then dehydrated gradually using increasing concentrations of ethanol (50, 70, 80, 85, 90, 95, and 100%). The ultrathin sections were prepared according to the standard protocol. Images were photographed using a transmission electron microscope (FEI Tecnai G^2^ 20 TWIN, United States).

### Total Protein Extraction

Bt cells grown in fermentation medium were harvested by centrifugation at 30 h. The cell pellets were washed three times with ice-cold PBS buffer (10 mM, pH 7.8) and then resuspended in 300 μl Lysis buffer containing 8 M urea, 2 M thiourea, 4% CHAPS (w/v), 75 mM NaCl, 50 mM Tris-HCl (pH 8.0), 5 μl PMSF, and 10 μl protease inhibitor cocktail. The suspension was further disrupted by sonication (3 s on, 3 s off) on ice for 10 min. Cell debris was removed by centrifugation at 13,200 rpm for at least 15 min, and the supernatant was transferred to another clean tube and stored at −80°C for further experiments. The concentrations of protein samples were determined using the Pierce^®^ BCA Protein Assay Kit following the manufacturer’s instructions. The quality of the protein samples extracted were checked by SDS-PAGE and the relative amounts of ICPs were analyzed using the Gel-Pro analyzer 4 software.

### Protein Digestion and iTRAQ Labeling

For each sample, 100 μg proteins were taken out and transferred into a new microcentrifuge tube. The proteins were solubilized and denatured by adding 100 μl Buffer I (8 M Urea, 0.1 M Tris-HCl, pH8.5) and 10 μl 10 mM DTT with incubation at 37°C for 2 h. The disulfide bond reformation was blocked by adding 10 μl 50 mM iodoacetamide and allowing to incubate in the dark for 15 min. Samples were then digested with 3 μg sequencing grade trypsin (Promega, United States) overnight at 37°C. The digested products were following collected and further subjected to a speed vacuum dry at 50°C for 3 h until completely dry. iTRAQ labeling was performed according to the manufacturer’s instruction. Briefly, the 8-plex iTRAQ reagents were firstly balanced to the room temperature, and then dissolved with 150 μl isopropanol. For each sample, a total of 100 μg peptides were differentially labeled with the iTRAQ reagents and incubated for 2 h at room temperature. The labeled tryptic digests were finally combined and lyophilized for further analysis.

### iTRAQ-Coupled 2D LC-MS/MS Analysis

Tryptic digest mixture was dissolved in 100 μl mobile phase A (10 mM ammonium formate in 98% water/2% acetonitrile, pH 10.0). The first dimensional separation at basic pH reverse phase was achieved on a 1200 Series HPLC (Agilent) containing a Zorbax Extend-C18 column (150 × 2.1 mm, Agilent). After a linear gradient elution with 5–50% mobile phase B (10 mM ammonium formate in 10% water/90% acetonitrile, pH 10.0), a total of 10 fractions from the first dimensional separation was collected and then lyophilized for further second dimensional online LC-MS analysis.

For LC-MS/MS analysis, the re-dissolved lyophilized SCX fractions were loaded into an Eksigent nanoLC-Ultra^TM^ 2D System (AB SCIEX, Concord, ON) containing a ChromXP C18 (3 μm, 120 Å) nanoLC trap column. The online trapping, desalting procedure were carried out with 100% solvent A (water/acetonitrile/formic acid: 98/2/0.1%). LC-MS/MS analysis was performed with a TripleTOF 5600 System (SCIEX, Concord, ON) fitted with a Nanospray III source. Data was acquired using an ion spray voltage of 2.4 kV, curtain gas of 30 PSI, nebulizer gas of 5 PSI, and an interface heater temperature of 150°C. The MS was operated with TOF-MS scans. For IDA, survey scans were acquired in 250 ms and as many as 30 product ion scans (80 ms) were collected if exceeding a threshold of 260 counts per second (counts/s) and with a + 2 to + 5 charge-state. A Rolling collision energy setting was applied to all precursor ions for collision-induced dissociation. Dynamic exclusion was set 16 s.

### Protein Identification and Quantification

The MS/MS data were converted into MGF format by AB SCIEX MS Data Converter 1.3 beta software and searched by three protein identification softwares (MyriMatch v2.2.8634, X!Tandem v2015.04.01.1, and MS-GF + v2016.06.29) through IPeak (Wen et al., 2015) against the *Bacillus thuringiensis* transcriptome database, which contained 6386 sequences. After MS/MS searching, proteins with at least one unique high confidence peptide and a false discover rate less than 1% were further quantified using IQuant software (Wen et al., 2014). Proteins with fold changes > 1.2 and *p* < 0.05 (Student’s *t*-test) were considered to be differentially expressed in the current study.

### Protein Annotation and Bioinformatic Analysis

The annotation of proteins identified were performed with Gene Ontology Terms^1^, COG Annotation^2^ and EggNOG Annotation^3^. The KEGG database^4^ was used to analyze the primary metabolic pathways and signal transduction pathways proteins involved in. Significantly enriched KEGG pathways were identified with a *p* < 0.05 in hypergeometric test as a threshold.

### Bioassays of Insecticidal Activity

Bt strains were grown in fermentation medium and in the LB medium to 60 h at 30°C, respectively. Appropriate volume of culture was harvested for each group and following subjected to a sonication process until cells lysed, if necessary. For bioassay, the artificial diet (100 ml medium containing 4 g yeast extraction, 7 g bean meal, 0.5 g vitamin C, 1.5 g agar, 1.5 ml 36% acetic acid and 2 g penicillin) were mixed with spore-crystal mixture at gradient concentrations for each group, and subsequently transferred to 24-well plates (Costar, United States). Each treatment was performed with three replicates and a total of 72 2nd-instar larvae were used per treatment. The negative controls were treated with blank culture medium. The plates were kept in a humidified growth chamber at 28°C with a 16:8 light: dark photo period. The mortality was recorded at 48 h post-incubation. The LC_50_ (50% lethal concentration) was calculated using Probit procedure of SPSS10.0 software.

## Results

### Construction of the *leuB* Gene Deletion Strain

**FIGURE 1:**
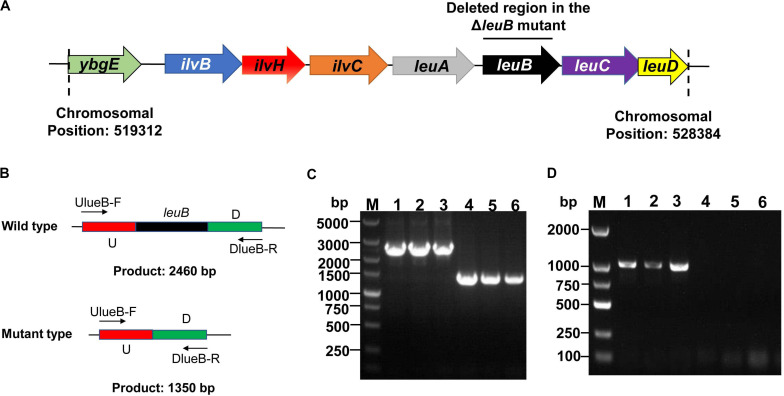
Construction of the *leuB* gene deletion strain. **(A)** Organization of the *ilv-leu* operon in Bt 4.0718. The DNA region within the *leuB* ORF deleted by the markerless gene deletion system is indicated. **(B)** Schematic of Δ*leuB* mutant type screening method using PCR analysis. U: upstream homologous arm, D: downstream homologous arm. **(C)** PCR confirmation of the *leuB* gene deletion using primers UleuB-F/DleuB-R. M, DNA marker; 1–3, Bt 4.0718; 4–6, Bt Δ*leuB* strain. **(D)** PCR confirmation of the *leuB* gene deletion using primers leuB-F/leuB-R. M, DNA marker; lane 1–3, Bt 4.0718; lane 4–6, Bt Δ*leuB* strain.

The genome sequencing and annotation results of Bt 4.0718 revealed that *leuB* gene was located in the *ilv-leu* operon which was involved in BCAA biosynthesis (Rang et al., 2015). The genetic organization of this *ilv-leu* operon in Bt 4.0718 was similar to that found in *Bacillus subtilis* (Figure 1A). The *leuB* gene of Bt 4.0718 was deleted by the I-SceI mediated markerless gene deletion system and confirmed by PCR and DNA sequence analysis. The resultant mutant was designated as Bt Δ*leuB* (Figures 1B–D).

### Bt Δ*leuB* Showed a Conditionally Sporulation-Deficient Phenotype

**FIGURE 2:**
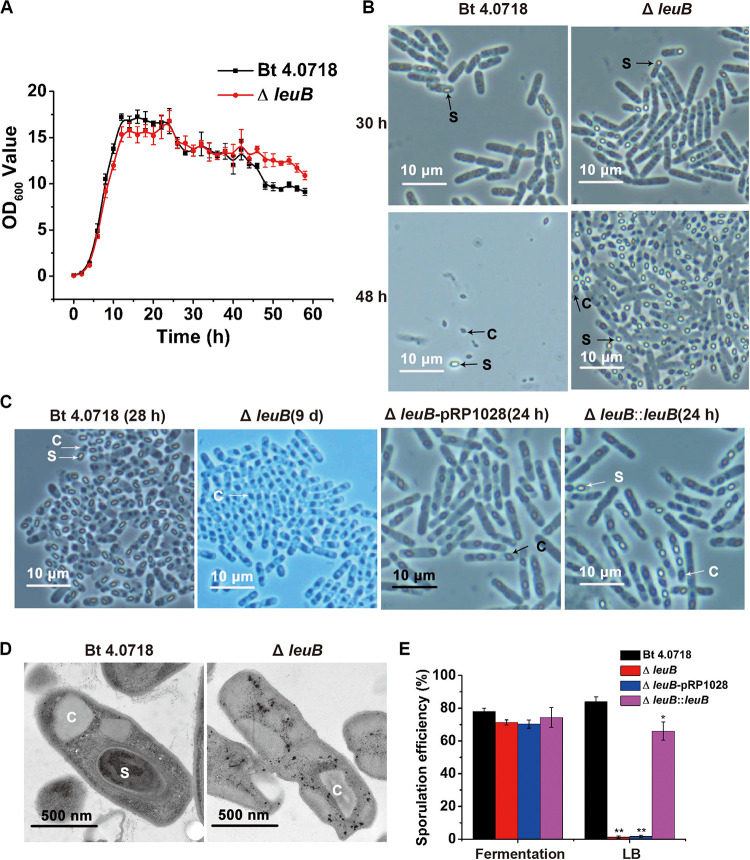
Phenotype characterization of Bt Δ*leuB* strain. **(A)** Growth curves of Bt 4.0718 and Bt Δ*leuB* strains in fermentation medium at 30°C. Data were mean values of three independent experiments (error bars are standard errors from mean values). **(B)** Morphology observation of Bt 4.0718 and Bt Δ*leuB* strain at time poins of 30 and 48 h in fermentation medium by phase-contrast microscopy. The spores and parasporal crystals are marked. C, parasporal crystals; S, spores. **(C)** Morphology observation of Bt 4.0718 and the mutant strains at time points indicated in LB medium by phase-contrast microscopy. **(D)** Transmission electron micrograph of Bt 4.0718 and Bt Δ*leuB* strain. **(E)** Comparison of sporulation efficiency of Bt 4.0718 and the mutant strains. Data were mean values of three independent experiments (error bars are standard errors from mean values). Significances of differences between Bt 4.0718 and the mutant strains were analyzed with SPSS by Student’s *t*-test. *P ≤ 0.05, **P ≤ 0.01 vs. Bt 4.0718 in LB.

Growth curves showed that deletion of *leuB* did not exert any inhibitory effect on the vegetative growth of Bt cells, and no obvious differences were observed at the early stage between these two strains in fermentation medium (Figure 2A). However, Bt Δ*leuB* showed a more prolonged stationary phase due to the delayed mother cell lysis as monitored by phase-contrast microscopy. The Bt 4.0718 mother cells had lysed and the spores and crystals are liberated at about 48 h, while most of Bt Δ*leuB* cells were found to contain encapsulated crystals and mature spores (Figure 2B) and a majority of the mutant cells didn’t autolyze until 58 h post-inoculation (data not shown). Moreover, the dimension of the spores in Bt Δ*leuB* were about 1.11 × 0.83 μm, which looked like smaller and rounder than that in Bt 4.0718 (1.55 × 0.85 μm) (Supplementary Figure S1). When cultured in LB liquid medium, Bt 4.0718 produced both spores and crystals, while Bt Δ*leuB* strain only produced crystal inclusions of great dimensions (Figure 2C), which was more evident under transmission electron microscopy (Figure 2D). Interestingly, it was found that the mother cells of the mutant did not lyse even after 9 days of growth, with crystals encapsulated within the cells. After complementation of Bt Δ*leuB* with plamid pRPleuB (Supplementary Figure S2), most cells of the complemented strain Bt Δ*leuB*:*leuB* sporulated normally in LB medium (Figure 2C).

The conditionally sporulation deficient phenotype of Bt Δ*leuB* was further validated by quantitative determination of the sporulation efficiency. No significant difference in sporulation efficiency was present for these strains in the fermentation medium. However, when cultured in LB medium, the sporulated cells were decreased to about 1% for Bt Δ*leuB* comparing to 84% for Bt 4.0718 (Figure 2E).

The sporulation phenotype of Bt Δ*leuB* was further investigated in two other glucose-free media: NSM and 1/2 LB liquid medium, respectively. Results showed that most Bt Δ*leuB* cells could produce mature spores after 20 h culture in these culture media (Supplementary Figure S3).

### Effect of *leuB* Deletion on ICPs Expression and Insecticidal Activity

**FIGURE 3:**
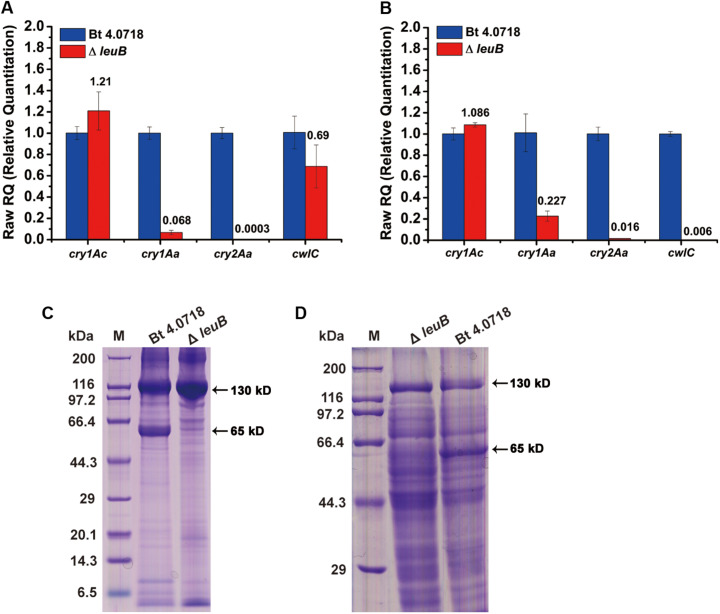
Effect of *leuB* deletion on *cry* gene transcription and ICPs production. **(A)** Relative expression levels of *cry1Ac*, *cry2Aa*, and *cwlC* gene in Bt 4.0718 and Bt Δ*leuB* strain at 24 h in fermentation medium were determined by qRT-PCR. Error bars are calculated from three independent experiments. **(B)** Relative expression levels of the three selected genes in Bt 4.0718 and Bt Δ*leuB* strain at 30 h in LB medium. **(C)** Effect of *leuB* deletion on ICPs production in fermentation medium detected by SDS-PAGE. The 130 and 65 kD ICPs bands were indicated by black arrows. Lane M, Protein marker. **(D)** Effect of *leuB* deletion on ICPs production in LB medium detected by SDS-PAGE. Lane M, Protein marker. The 65 kD ICPs band of Bt 4.0718 was further confirmed by 1D-LC-MS/MS analysis (Supplementary Table S2).

Although Bt Δ*leuB* showed a conditionally asporogenous phenotype, it produced large bipyramidal crystals in two types of culture medium. The parasporal crystals of Bt 4.0718 were mainly composed of two protoxins: Cry1Ac and Cry2Aa (Song et al., 2005; Sun et al., 2008). As shown in Figures 3A,B, comparing to Bt 4.0718, except that *cry1Ac* was increased, *cry2Aa* and *cwlC* in Bt Δ*leuB* were all significantly down-regulated at the transcriptional level in both culture conditions. CwlC is a cell wall hydrolase newly characterized, which is essential for Bt mother cell lysis (Chen et al., 2018). Interestingly, consistent with the blocked mother cell lysis of Bt Δ*leuB* in LB medium, the relative mRNA level of *cwlC* was also decreased to an extreme low degree (Figure 3B).

Meanwhile, the relative amounts of ICPs produced by two strains were determined by SDS-PAGE. The amount of 130 kDa ICPs in Bt Δ*leuB* was increased (fermentation medium) or basically identical (LB) comparing to that in Bt 4.0718 strain, while the 65 kDa ICPs levels were significantly decreased in both media (Figures 3C,D and Supplementary Table S2). To determine the insecticidal activities of these two strains, laboratory bioassay against *Plutella xylostell* and *Helicoverpa armigera* larvae were performed. Results showed that no significant differences in the LC_50_ values between these two strains in every test group were determined, suggesting that deletion of *leuB* had little negative effect on insecticidal activity toward these two target insects (Table 2).

**TABLE 2:** Insecticidal activities of Bt 4.0718 and Bt Δ*leuB* strains against *H. armigera* and *P. xylostell.*

Insect species	Medium	lg (LC_50_ μl/ml)	
		Bt 4.0718 Bt Δ*leuB*	
*H. armigera*	Fermentation	2.86 (2.75–3.02)^a^	2.03 (1.81–2.59)
	LB	3.01 (2.82–3.23)	2.2 (1.95–2.30)
*P. xylostell*	Fermentation	1.05 (0.89–1.15)	1.01 (0.81–1.15)
	LB	1.44 (1.0–1.63)	1.04 (0.81–1.20)

*^*a*^The 95% confidence intervals were indicated in parentheses. Each mean value was calculated from three replicate experiments.*

### iTRAQ-Based Comparative Proteomic Analysis Between Bt 4.0718 and Bt Δ*leuB* Strain

**FIGURE 4:**
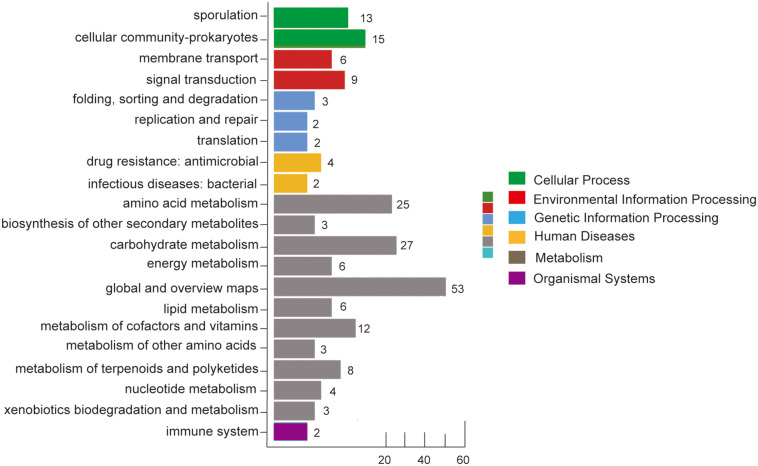
Functional classification of differentially expressed proteins (DEPs) caused by *leuB* deletion. DEPs were categorized into functional categories according to GO and KEGG pathway analysis.

To obtain a global view of how the Bt cell metabolic network adjusts to deletion of its *leuB* gene, an iTRAQ-based comparative proteomic analysis between these two strains was performed. Strains were both cultured in the fermentation medium, and cells grown to the mid-stationary phase (30 h) were collected separately. By using iTRAQ-coupled 2D LC-MS/MS approach, a total of 2821 proteins were identified from three biological replicates (Details for each protein identified were provided in Supplementary Table S3). The number corresponded to 41.8% of the predicted Bt 4.0718 proteome. However, CwlC wasn’t located in the list of proteins identified. The relative quantification data of each of the identified proteins was obtained, and those having a fold change > 1.2 and *p* < 0.05 were considered as differentially expressed proteins (DEPs). Comparing to Bt 4.0718, 61 proteins increased and 145 decreased in Bt Δ*leuB*, among which Cry 1Ac and Cry2Aa were identified with fold change of 1.45 and 0.33, respectively (Table 3). The DEPs were classified into functional categories according to the GO and KEGG pathway annotations. Results revealed that DEPs response to *leuB* deletion were mainly enriched in sporulation, amino acid metabolic process and carbohydrate metabolism (Figure 4), which indicated adaptation of the Bt proteome to deletion of its *leuB* gene.

**TABLE 3:** Differential expressed proteins mentioned in the discussion.

Protein ID	Description	Fold change
A1E258	Pesticidal crystal protein Cry1Ac	1.45
A0A0A7RCB9	Pesticidal crystal protein Cry2Aa	0.33
A0A231I419	3-hydroxybutyrate dehydrogenase	1.22
A0A0G3DWY1	(R, R)-butanediol dehydrogenase	1.29
A0A0G3EBM1	Acyl-CoA carboxylase	1.24
A0A0G3E5D6	Acyl-CoA dehydrogenase	1.21
A0A2T7YXZ2	Phosphoenolpyruvate carboxykinase	0.81
A0A0B5XJI8	L-lactate dehydrogenase	0.83
C3DNK2	DPA synthetase B subunit	0.56
A0A242ZWY3	Dipicolinate synthase subunit A	0.67
A0A1B1LGA7	Homoserine dehydrogenase	1.30
W8Y155	Ketol-acid reductoisomerase	1.28
C3H608	Leucine dehydrogenase	1.29
C3GH78	BACC aminotransferase	1.35
C3HBF2	Asparagine synthetase B	1.24
A0A243G9N6	O-acetylhomoserine amino carboxypropyl transferase	1.33
A0A1B1T3W7	ABC transporter substrate-binding protein	1.43
A0A2T7YQX7	ABC transporter permease	1.27
A0A0N8VH35	SpoIIIJ	0.68
C3DT56	SpoIIID	0.83
A0A0N8VHF0	dTDP-glucose pyrophosphorylase	0.80
A0A243G461	dTDP-4-dehydrorhamnose reductase	0.73
A0A243G2H3	Spore cortex protein	0.78
A0A0Q0RCX0	Spore coat protein CotJC	0.80
C3DSD2	Spore coat protein CotF	0.59
A0A0G3E1Y2	Spore coat protein CotY	0.66
A0A0G3E3Y1	Spore coat protein CotE	0.64
A0A243NJJ2	Spore coat protein GerQ	0.81
A0A0S1TRK3	Spore coat-associated protein N	0.83
A0A0G3E429	Stage V sporulation protein T	0.81
A0A0G3DZL1	Sporulation protein YtfJ	0.79

### The Metabolic Responses of Bt 4.0718 to Deletion of Its *leuB* Gene

We mainly focused on DEPs closely involved in spore and ICPs formation in Bt (Table 3). They were classified into the following categories: carbon flux and utilization, amino acid synthesis and transport, and spore formation related proteins.

#### Carbon Flux and Utilization

It is generally believed that the carbon-energy storage substances, such as fatty acid, acetoin and poly-3-hydroxybutyrate (PHB), play a critical role in energy supply for the spores and ICPs formation process in Bt (Wang et al., 2013), although a recent study led by Wang et al. (2016) pointed out that PHB metabolism was unrelated to sporulation and ICPs synthesis. We found that PHB utilization-related protein 3-hydroxybutyrate dehydrogenase (Hbd), acetoin biosynthesis-related protein (R, R)-butanediol dehydrogenase (ButB), fatty acid biosynthesis or degradation-related protein Acyl-CoA carboxylase and Acyl-CoA dehydrogenase were all identified and increased in Bt Δ*leuB*. These results indicated that compared to Bt 4.0718, more carbon source fluxed into these carbon-energy storage substances during the logarithmic growth phase in the mutant.

During the sporulation process, pyruvate is mainly generated from oxaloacetate, lactate and odd chain fatty acid via gluconeogenesis, and would be used as the biosynthetic precursor of DPA, a major component in the spore core (Orsburn et al., 2010; Wang et al., 2013). In Bt Δ*leuB*, phosphoenolpyruvate carboxykinase – the key enzyme converting oxaloacetate to pyruvate, L-lactate dehydrogenase, and even both of the DPA synthetase subunit A and B were found significantly decreased. It was indicated the supply of pyruvate and synthesis of DPA might be down-regulated in the mutant, which may exert some impact on sporulation.

#### Amino Acid Synthesis and Transport

Quantitative data reveals that homoserine dehydrogenase, ketol-acid reductoisomerase, leucine dehydrogenase and BCAA aminotransferase involved in BCAA biosynthesis, asparagine synthetase B and O-acetylhomoserine amino carboxypropyl transferase, as well as the amino acid transport-related proteins such as ABC transporter substrate-binding protein and ABC transporter permease were all increased in Bt Δ*leuB*. It suggested that both intracellular synthesis and absorption of amino acids were strengthened in the mutant.

#### Spore Formation Related Proteins

It was found that the spore formation-related transcriptional regulators σ^H^, SpoIIIJ and SpoIIID, the spore germ cell wall biogenesis-related dTDP-glucose pyrophosphorylase and dTDP-4-dehydrorhamnose reductase, spore cortex protein, spore coat-associated protein N, stage V sporulation protein T, sporulation protein YtfJ, together with spore coat protein CotJC, CotF, Cot Y, CotE, and GerQ were all down-regulated.

**FIGURE 5:**
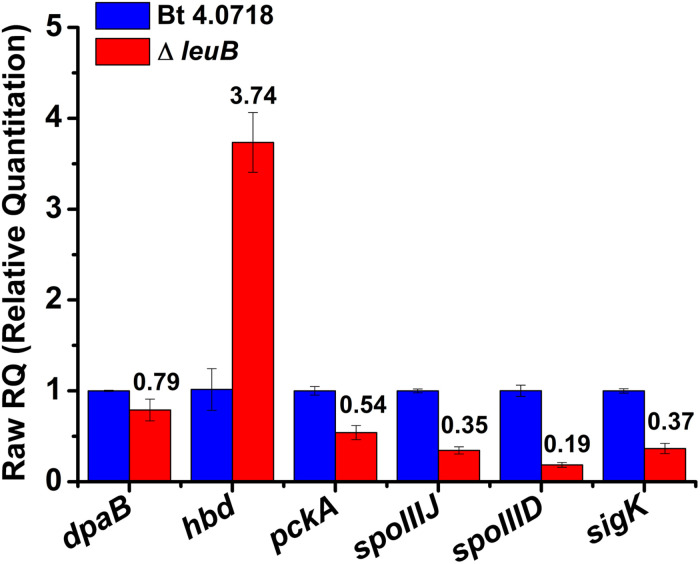
Relative expression levels of the *dpaB*, *hbd*, *pckA*, *spoIIIJ*, *spoIIID*, and *sigK* genes in Bt strains at 30 h in fermentation medium determined by qRT-PCR. *dpaB*, encoding for DPA synthetase B subunit; *hbd*, 3-hydroxybutyrate dehydrogenase; *pckA*, phosphoenolpyruvate carboxykinase.

Five representative DEPs: DPA synthetase B subunit, 3-hydroxybutyrate dehydrogenase, SpoIIIJ and SpoIIID, together with the σ^K^ factor were subjected to transcriptional level analysis. qRT-PCR results showed that the expression patterns of mRNA were consistent with those of protein, which indicated that the DEPs analysis were reliable (Figure 5).

### Limitation of Pyruvate (Carbon) Supply Is Responsible for the Conditionally Asporogenous Phenotype of Bt Δ*leuB*

**FIGURE 6:**
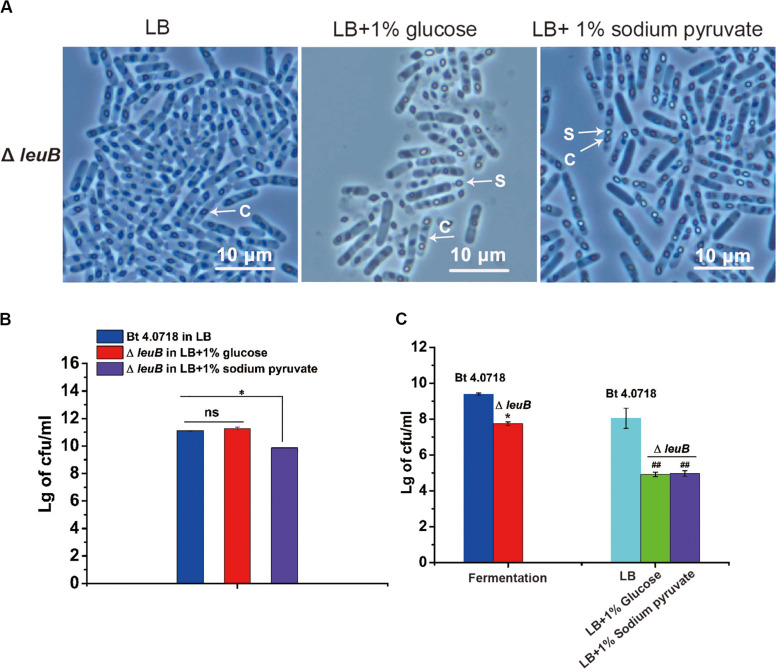
Limitation of pyruvate (carbon source) supply is responsible for the conditionally asporogenous phenotype of Bt Δ*leuB*. **(A)** The asporogenous phenotype of Bt Δ*leuB* in LB medium was rescued by supplementing 1% glucose or 1% sodium pyruvate. The sporulation was observed by phase-contrast microscopy at 60 h post inoculation. **(B)** Spores were quantified by counting heat resistant CFUs in LB + 1% glucose and LB + 1% sodium pyruvate group, which were compared with that of Bt 4.0718 in LB medium. ^ns^*P* > 0.05; *P ≤ 0.05. **(C)** The relative heat-resistance potency (90°C, 15 min) of each group in all culture conditions were quantified. *P ≤ 0.05 vs. Bt 4.0718 in fermentation medium, ^##^*P* ≤ 0.01 vs. Bt 4.0718 in LB medium.

The carbon source, nitrogen source and carbon/nitrogen (C/N) ratio might play an important role on *Bacillus* sporulation (Oh et al., 1995). The C/N ratio of 1/2 LB medium was the same as that of LB, however, its carbon source concentration was half of that in LB medium. Based on results determined in Supplementary Figure S4, the cell concentration of Bt Δ*leuB* in LB was 1.30 × 10^11^ cfu/ml comparing to 4.86 × 10^10^ cfu/ml in 1/2 LB medium, the carbon source acquired by each mutant bacterial cell was much lower in LB medium than that in 1/2 LB medium. Combined with the quantitative proteomics data, it was suggested that the inhibited supply of pyruvate (carbon source) is an important factor resulting in the conditionally asporogenous phenotype of Bt Δ*leuB*. It was speculated that the asporogenous phenotype in LB medium would be corrected by supplementing with pyruvate or glucose (converted to pyruvate via EMP pathway). Consistently, the results of phase-contrast microscopy observation showed that this asporogenous phenotype of the mutant in LB medium could be rescued by supplementing 1% glucose or sodium pyruvate (Figure 6A). However, in the 1% sodium pyruvate added group, the number of sporulated cells was obviously lower than that of Bt 4.0718 strain and the 1% glucose added group (Figure 6B). Moreover, the spores of Bt Δ*leuB* in both fermentation medium and supplemented LB medium showed a significantly reduced resistance to heat treatment (90°C) comparing to that of Bt 4.0718 (Figure 6C).

## Discussion

According to the existing literature, sunlight-triggered inactivation of Bt crystals is often cited as the major factor affecting the pest-control duration of Bt products (Pusztai et al., 1991; Demuth et al., 2015). Here, we report the construction of a new recombinant Bt strain with delayed or completely blocked mother cell lysis. We demonstrated for the first time that LeuB was not only an enzyme directly involved in leucine biosynthesis, but also an important functional gene affecting the formation of spores and ICPs in Bt.

The most intriguing feature of Bt Δ*leuB* is its two different sporulation phenotypes, depending on carbon source supply. It showed a marked decrease in spore size while a significantly improvement of ICPs production in fermentation medium (Supplementary Figure S1 and Figure 3C). The overproduction of ICPs in Bt Δ*leuB* can be mainly attributed to the prolonged stationary phase as a consequence of delayed cell lysis which would allow additional time for Cry proteins accumulation. Moreover, when cultured in LB liquid medium, the Bt Δ*leuB* cells were unable to sporulate while produced larger parasporal crystals that remained encapsulated within the mother cells (Figures 2C–E). It seemed that the block to sporulation here was downstream from σ^E^ in the regulatory cascade. That is because at least σ^E^ is required for supporting the high level transcription of *cry1* gene (Bravo et al., 1996). It was worth noting that the σ^K^-dependent cell wall hydrolase encoding-gene *cwlC* transcription of the mutant was found to be down-regulated in both culture media, especially severely decreased in LB medium, which resulted in a delay or block of mother cells. The previous study conducted by Sanchis has demonstrated that this encapsulated crystals can reduce the loss of toxity under UV irradiation (Sanchis et al., 1999). Interestingly, qRT-PCR results revealed that only *cry1Ac* transcription was slightly increased, while *cry2Aa* was severely decreased in both types of media and the underlying mechanism might be complex (Figure 3). Because the expression of *cry* genes is regulated in a more sophisticated way than we think. In addition to σ^E^ and σ^K^ factors, they can also be regulated through mechanisms at various levels. Although a decreased toxicity to *H. armigera* and *P. xylostell* (susceptible to Cry1Ac) was not observed in the mutant, its loss of the protoxin Cry2Aa might exert some negative impact on the insecticidal spectrum of this mutant strain.

Although LeuB was identified as a limiting factor for biosynthesis of Leucine, *leuB* deletion did not exert any inhibitory effect on the vegetative growth and crystals formation of Bt cells in these culture media. It was speculated that cells might overcome this metabolic bottleneck through other pathways such as uptaking Leucine from culture medium and protein recycling (Monro, 1961). The role of LeuB during sporulation could be important, as this enzyme has a broad specificity and is able to utilize malate as substrates in addition to 3-isopropylmalate (Kawaguchi et al., 1993; Drevland et al., 2007). It has been reported that the expression of malate dehydrogenase citH is markedly down-regulated during sporulation, and LeuB would be likely recruited to catalyze malate dehydrogenation in the final TCA cycle step (Wang et al., 2013). Therefore, the present study suggests that *leuB* gene deletion can inhibited the direct conversion of malate to oxaloacetate. As is known to all, during sporulation, oxaloacetate is likely a major source of pyruvate (Wang et al., 2013), and the demand for pyruvate in cell has been increasing because of the high-level synthesis of DPA. Quantitative proteomics results showed that expression of phosphoenolpyruvate carboxykinase and both of the DPA synthetase subunit A and B were all significantly decreased in Bt Δ*leuB* (Table 3). DPA is synthesized in the mother cell during late stage sporulation and comprises about 15% of the spore dry weight (Orsburn et al., 2010; Setlow et al., 2017). In *Bacillus* species, pyruvate is the key precursor for DPA biosynthetic pathway. Pyruvate and aspartate semialdehyde are first condensed to produce dihydrodipicolinic acid (DHDPA), and DHDPA is further converted to DPA by the DPA synthase (Daniel and Errington, 1993; Orsburn et al., 2010). In fermentation medium, the decreased synthesis of DPA might exert some impact on the heat resistance property of spores in Bt Δ*leuB*. The sporulation phenotype of Bt Δ*leuB* in LB medium, however, was quite different from that in two other glucose free media (1/2 LB and NSM media). It was speculated that the carbon source acquired by each Bt Δ*leuB* cell was decreased to a much lower level in LB medium, which finally resulted in its asporogenous character. Interestingly, we showed that the addition of 1% sodium pyruvate and in particular 1% glucose could restore sporulation to Bt Δ*leuB* strain (Figure 6). However, probably because of less DPA supplied as indicated by aforementioned data, these smaller spores produced by Bt Δ*leuB* showed a greatly decreased resistance to heat treatment (90°C) comparing to that of Bt 4.0718 (Figure 6C).

On the other hand, consistent with the decreased spore size, deletion of *leuB* also resulted in decreased expression of sporulation-related transcriptional regulators SpoIIIJ and SpoIIID, as well as a series of spore structural proteins (Table 3). In *Bacillus subtilis*, expression of *spoIIIJ* occurs during vegetative growth and last until sporulation, and is essential for the activation of σ^G^ and for sporulation (Errington et al., 1992; Serrano et al., 2003; Geng et al., 2014). SpoIIID is a sequence-specific DNA-binding protein that mainly involved in transcriptional activation or repression of many σ^E^ or σ^k^-directed genes. In *Bacillus subtilis* and *Clostridium difficile*, *spoIIID* is induced by σ^E^ factor, and it positively regulates transcription of σ^k^ factor (Wang et al., 2007; Pishdadian et al., 2015). Therefore, the down-regulation of sigma factors late in sporulation such as σ^G^ and σ^k^ may account for changes in the expression of spore structural proteins.

**FIGURE 7:**
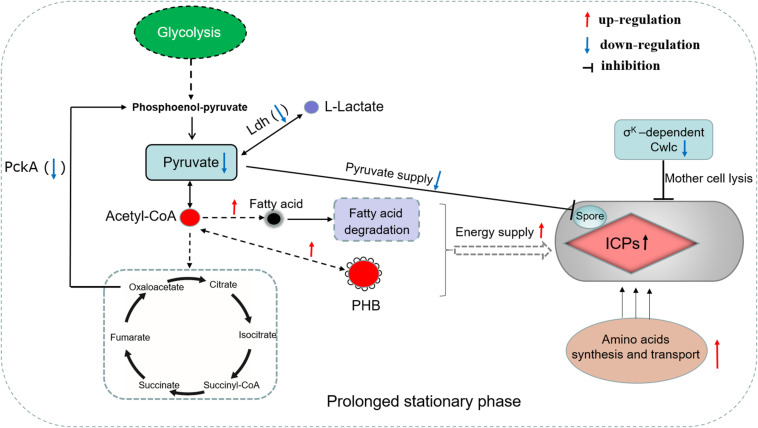
Proposed metabolic changes associated with spore development, crystal protein formation and mother cell lysis of Bt cell due to deletion of its *leuB* gene. PckA, phosphoenolpyruvate carboxykinase; Ldh, L-lactate dehydrogenase; PHB, poly-3-hydroxybutyrate.

Additionally, the mother cell lysis phenotype is always coupled with sporulation because of the σ^k^-dependent CwlC (Chen et al., 2018). In this study, the σ^k^ and CwlC down-regulation in Bt Δ*leuB* resulted a considerable delay of cell lysis in the fermentation medium, which provided additional time for ICPs synthesis. How to adjust to the high energy, carbon sources and amino acids consumption brought about by increased production of ICPs is a question for the cell machinery. Consequently, our proteomics results successfully revealed the metabolic adaptations employed by the cells under this specific condition. A series of DEPs involved in utilization of the carbon-energy storage substances such as PHB, acetoin and fatty acid were up-regulated, which indicated that more carbon source fluxed into these substances in the mutant during the logarithmic growth phase (Grundy et al., 1993; Navarro et al., 2006; Wang et al., 2013). And these stored carbon substances would be re-utilized for ICPs synthesis under nutrient-deficient stationary phase. Moreover, amino acid synthesis and transport activities were also enhanced in the mutant strain, which provided sufficient amino acids precursors for high-level protein synthesis (Figure 7).

In summary, as LeuB fulfills an unusual role in the final TCA cycle step during sporulation, *leuB* deletion caused a disturbance of the metabolic balance in sporulating cells, which finally resulted in the conditionally asporogenous feature of this strain. Although the expression of some *cry* genes is reduced in the *leuB* mutant which may decrease its toxicity for certain insects, the overexpression of Cry1Ac (or other Cry proteins that remains to be verified in the future) and delayed or blocked mother cell lysis make it somewhat an attracting recombinant strain for future application. More importantly, this information provides us with novel insight into the metabolic node points associated with sporulation of Bt, which may be useful for further strain improvement.

## Data Availability Statement

All datasets generated for this study are included in the article/Supplementary Material.

## Author Contributions

MQ, JP, and XD conceived and designed the research. MQ, JP, ZZ, SL, JX, and PZ conducted the experiments. LX and YS analyzed the data. MQ wrote the manuscript. All authors made suggestions to the manuscript revision and approved the final version.

## Conflict of Interest

The authors declare that the research was conducted in the absence of any commercial or financial relationships that could be construed as a potential conflict of interest.

## Footnotes

1 http://www.geneontology.org/

2 http://www.ncbi.nlm.nih.gov/COG/

3 http://eggnogdb.embl.de/

4 https://www.kegg.jp/
