# Supplementary material for: Construction of a Conditionally Asporogenous Bacillus thuringiensis Recombinant Strain Overproducing Cry Protein by Deletion of the leuB Gene
Source: Front Microbiol. 2020 Jul 24;11:1769. doi: 10.3389/fmicb.2020.01769 (PMC7396631; doi:10.3389/fmicb.2020.01769)
Supplement: Supplementary file 1 [file Table_1.docx]

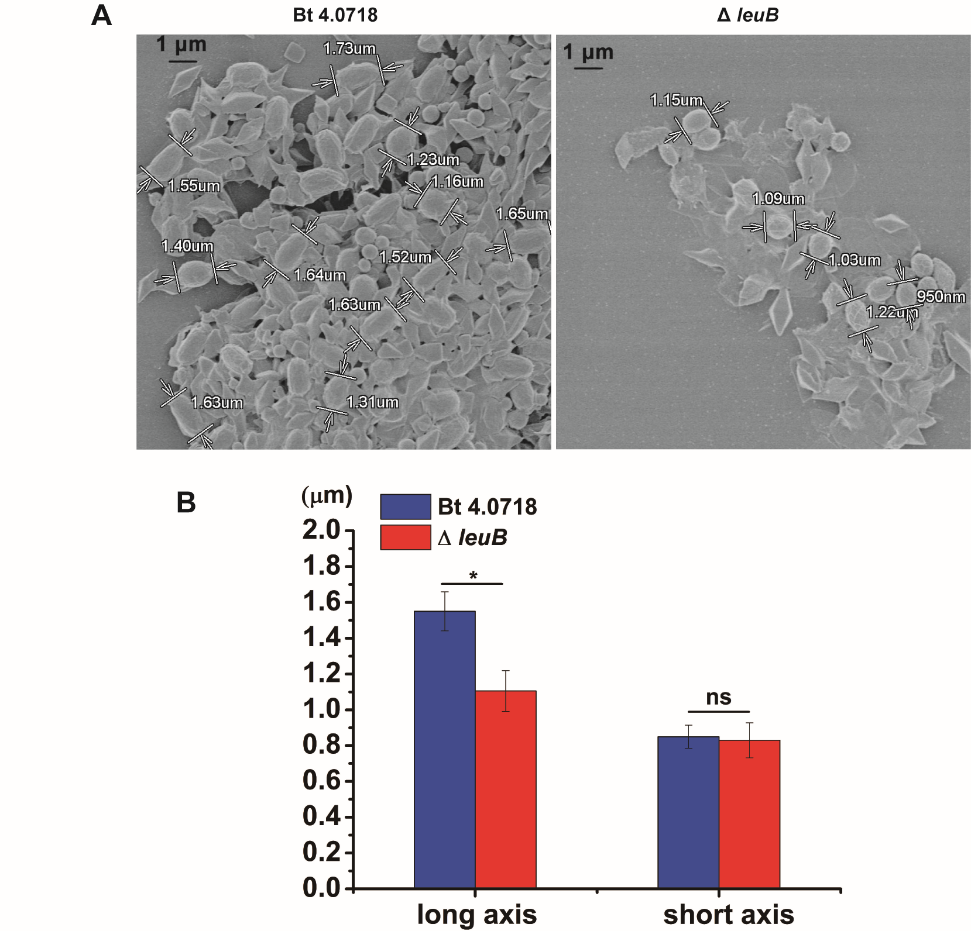


**FIGURE S1 Scanning electron micrograph of spores produced by Bt 4.0718 and Bt Δ*leuB*. (A)** Electron micrograph and the dimensions of the spores measured. The mixtures of crystals and spores were harvested from cultures with the fermentation medium. The long axis sizes of some spores selected randomly were indicated. **(B)** Quantification of the spore sizes in these two strains. Altogether, about 50 cells from each strain were measured. Data were presented as means ± standard errors. Signiﬁcances of differences were analyzed with SPSS by Student’s t-test. ^ns^*P* > 0.05, ^*^*P* ≤ 0.05.


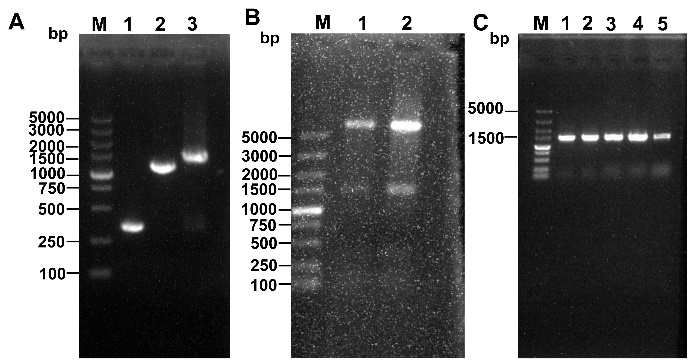


**FIGURE S2 Construction of the Bt Δ*leuB* complementation strain. (A)** PCR amplification of the *Perm*, *leuB* ORF and *Perm*-*leuB* fragments. M: DNA marker, 1: *Perm*, 2: *leuB* ORF, 3: *Perm*-*leuB* fragment. **(B)** Double digestion confirmation of plasmid pRPleuB. M: DNA marker, 1-2: *Bam*H I/*Sal* I digestion of pRPleuB. **(C)** Confirmation of the complemented strain Bt Δ*leuB*::*leuB* using PCR. M: DNA marker, 1-5: PCR confirmation using primers P-F/E-R.


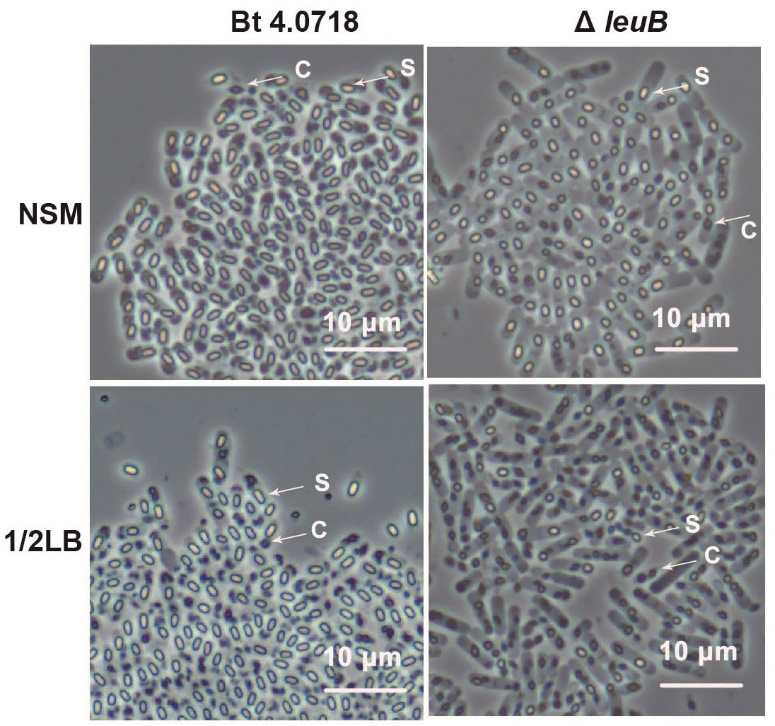


**FIGURE S3 Sporulation phenotype characterization of Bt Δ*leuB* strain in NSM and 1/2 LB media.** Morphology observation of Bt Δ*leuB* strain at 20 h in NSM and 1/2 LB media by phase-contrast microscopy. The spores and parasporal crystals are marked. C: parasporal crystals, S: spores.


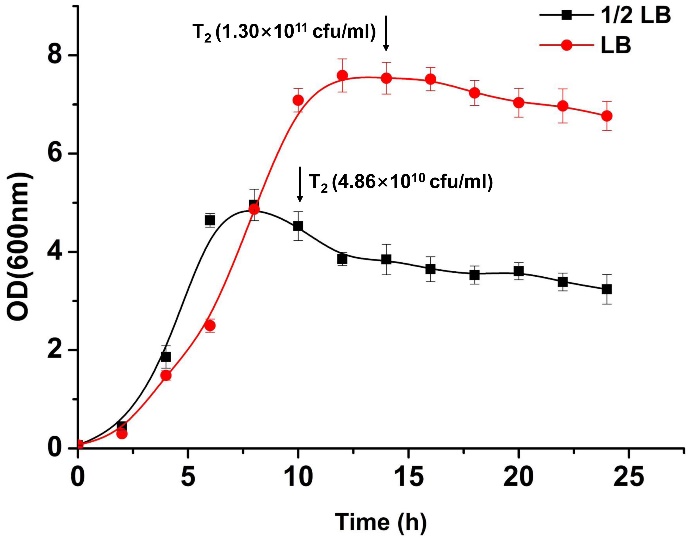


**FIGURE S4 Growth curves of Bt Δ*leuB* in 1/2 LB and LB media at 30℃.** The number of vegetative cells per milliliter at T_2_ was estimated by counting the colony-forming units (CFUs) and were indicated by black arrows.

**TABLE** **S1** Primers used in this study

| **Prime** | **Sequence (5'–3')** | **Restriction site** |
| --- | --- | --- |
| UleuB-F | CGCGGATCCCATTTGCTCATGAATCGGG | *Bam*H I |
| UleuB-R | ACGCGTCGACGGTGAGGAAAGCTTCTCTCA | *Sal* I |
| DleuB-F | ACGCGTCGAC ATGGGGAAAAGGTTACTAGA | *Sal* I |
| DleuB-R | CGGGGTACC AAAGCGTCATCCTCTCTTC | *Kpn* I |
| leuB-F | CTAGCTAGCATGGAAAAACGTATCGTTTGTTTAG |  |
| leuB-R | CCGCTCGAGTCTTCCTCTCCCTACTAACGCT |  |
| P-F | CGCGGATCCTGATCCCCCTTAGAAGCAA | *Bam*H I |
| P-R | GTAATTACTCCTGAAGTGATTACAT |  |
| E-F | ATTTACAGATGTAATCACTTCAGGAGTAATTACATGGAAAAACGTATCGTTTGTT |  |
| E-R | ACGCGTCGACTCCAATCCATTTTCATTAGTCG | *Sal* I |
| cry1Ac-F | ATATTTCCTTGTCGCTAACGCA |  |
| cry1Ac-R | TGTACAAGAAATGCGTCCCATT |  |
| cry2Aa-F | CCCTTGCTCGTGTAAATGCA |  |
| cry2Aa-R | AGGAACAGGGTTTTGAGTAGGG |  |
| cwlC-F | AAGTTAAAGATGCCGTGGCG |  |
| cwlC-R | CAGCATGGGAATTGCTATTCC |  |
| dpaB-F | GGGAGCAGAAGTACGTCCTGTT |  |
| dpaB-R | CCACTCTGCTCCTTCTCCAAA |  |
| hbd-F | GTTGTGATTACCGATCGACTTGA |  |
| hbd-R | GCTGCTGTAATTTCCTCTTCAGA |  |
| pckA-F | TGTGAACCAACCGATTTCTGAA |  |
| pckA-R | TGCAAATCCTTTGAAGACGAAT |  |
| spoⅢJ-F | GTTGCTCATCATATACCGGGAG |  |
| spoⅢJ-R | CTGCGATATTGCGAAACAGC |  |
| spoⅢD-F | CCAGAAATTAATCCAGAGCTCG |  |
| spoⅢD-R | GTTGCTTCTCCCCCTCTTAAAT |  |
| sigK-F | AGTTAATGGAGCAAGGTGATGC |  |
| sigK-R | CTGCATCTTCTCCTGTGTTCTCA |  |
| 16S-F | CTTGACATCCTCTGAAAACCCTA |  |
| 16S-R | GACTTAACCCAACATCTCACGAC |  |

**TABLE S2** **Protein identification by 1D-LC-MS/MS analysis^a^**

| **Accession** | **Unique Peptides** | **MW (kDa)** | **Description** | **Score** |
| --- | --- | --- | --- | --- |
| P0A377 | 12 | 70.8 | Pesticidal crystal protein Cry2Aa | 869.93 |

^a^ The 65 kD ICPs band of Bt 4.0718 in Fig.3D was confirmed by 1D-LC-MS/MS analysis.
